# Weight cutting in female UFC fighters

**DOI:** 10.1080/15502783.2023.2247384

**Published:** 2023-08-24

**Authors:** Cassandra Evans, Charles Stull, Gabriel Sanders, Anthony Ricci, Duncan French, Jose Antonio, Corey A. Peacock

**Affiliations:** a Nova Southeastern University, Department of Health and Human Performance, Fight Science Lab, Fort Lauderdale, FL, USA; b UFC Performance Institute, Las Vegas, NV, USA; c Northern Kentucky University, Department of Kinesiology and Health, Highland Heights, KY, USA; d Australia Catholic University, School of Health Sciences, Melbourne, Australia; e Edith Cowan University, School of Medical and Health Sciences, Perth, WA, Australia

**Keywords:** Weight cuts, weight loss, female athletes, mixed martial arts

## Abstract

**Background:**

It is common practice for fight sport athletes to use a variety of weight manipulation strategies to compete in desired weight classes. Although numerous studies have highlighted rapid weight loss (RWL) strategies and the magnitude of weight loss, few have focused specifically on weight loss in female fighters. The purpose of this study was to provide descriptive information on professional UFC female fighters engaging in RWL in all women’s UFC weight divisions: strawweight (52.2 kg): flyweight (56.7 kg); bantamweight (61.2 kg); featherweight (65.8 kg).

**Methods:**

All fighter’s weights were obtained at five separate time points: 72 hrs. pre-weigh-in, 48 hrs. pre-weigh-in, 24hrs. pre-weigh-in, official weigh-in, and 24 hrs. post-weigh-in (competition weight). Mixed effects models and random effects analysis were used to assess changes in weight and differences between weight divisions. All statistics were analyzed, and significance was set at *p* ≤0.05. Significant changes in weight between all time points were reported.

**Results:**

No statistical differences between weight divisions were observed. Female fighters lost 4.5–6.6% of their weight prior to the official weigh-in.

**Conclusion:**

Females engaged in RWL practices lose weight in a similar fashion irrespective of weight class.

## 1. Introduction

The most recognized fight sport is mixed martial arts (MMA) which utilizes fighting techniques such as striking, kicking, and grappling [1]. MMA made its initial debut in the public eye during the early 1990s and was catapulted into mainstream sports via the Ultimate Fighting Championship (UFC) [2–4]. In the early days of the UFC, few rules existed. Fights ended via knockouts, submission, or abandonment with no limit on time [3,4]. Over the years, the UFC added rules and regulations to the organization transforming it into a global force [3,4]. The UFC is considered the premier professional MMA organization [4]. Today, athletes competing in the UFC are divided into categories based on weight and gender. Female UFC fighters compete in 1 of 4 weight divisions: strawweight (115 lbs/52.2 kg): flyweight (125 lbs/56.7 kg); bantamweight (135 lbs/61.2 kg); featherweight (145 lbs/65.8 kg) [5].

With the creation of weight divisions, it is a common practice for fighters to compete in weight divisions lower than their usual body weight (UBW) in an attempt to gain a competitive size advantage. Fight sports are characterized by explosive movements requiring the athlete to generate significant force at high velocities. This makes lean body mass (LBM) particularly important due to its influence on relative force production and the rate of force production [6,7]. Based on this, it has been posited that the greater the lean mass of an athlete, the greater the rate of force that can be produced during a kick or punch at a given weight. Unlike gradual weight loss, “making weight” is conducted over a short period of time. It is not uncommon for fighters to lose 7% of their body weight within days of the official weigh-in [8–13]. These fighters will typically gain as much as 10% of their weight back by competition [9]. It is important to note that weight loss within 72 hours of competition is typically due to transient changes in body fluid as opposed to sustained changes in body mass [14]. LBM retains more water compared to adipose tissue allowing for a greater amount of weight loss to occur from water manipulation [14–17]. Contrary to popular belief, the literature surrounding the health effects of RWL, in both field and laboratory, is mixed [14,18–20]. Although deleterious effects of dehydration are well documented, fight sports generally designate a specific window of time post-weigh-in for rehydration. Proper recovery restores an athlete at or near their ideal performance weight, minimizing the negative impact on performance [18]. Thus, fighters who choose to compete in a lower weight class may potentially gain a physical advantage due to the maintenance of explosive strength and power compared to smaller opponents. Furthermore, fighters are less likely to experience performance decrements related to RWL using methods such as water manipulation and moderate dietary restriction as opposed to more aggressive practices (diuretics, laxatives, and vomiting) [18,19].

Numerous strategies are employed to induce rapid weight loss (RWL) such as calorie restriction, water manipulation, and exercise [5,19,21,22]. Water loading with subsequent daily reductions is a common practice among MMA fighters. Large volumes of water are ingested followed by restricted intake. This hyperhydrated state triggers a hormonal response in the renal system that results in increased fluid output [16,19]. Restricting the intake of specific nutrients like carbohydrates, dietary fibers, and sodium contributes to water manipulation. When manipulating gut contents, reducing total daily intake is preferred over the use of harsh laxatives [18]. Water manipulation combined with modifying dietary intake is thought to be a safe and effective method for making weight [14,16,19]. Following specific, predetermined, RWL protocols may help athletes make weight with greater safety and potentially decrease negative effects.

Much has been written about the physiological response, performance outcomes, and implications of weight cuts in male fight sports [5,19,21,22]. There is scant literature detailing the response, success, and implications of RWL in female fighters. The physiological and morphological differences between male and females is well accepted. Many recommendations are based on these gender differences. For example, the minimum acceptable body fat (BF) percentage for women is 12% and 5% for men [23,24]. Furthermore, the menstrual cycle induces changes (physiologically and psychologically) that may influence sports performance and weight cutting [24–26]. Menstrual cycles vary among females. Generally, they are separated by four distinct phases: menstruation, follicular phase, ovulation, and the luteal phase [27–29]. Each phase is characterized by different fluctuations in hormones such as progesterone and estrogen [27–29]. It is not uncommon for female athletes to experience irregular periods (oligomenorrhea) or the absence of the menstrual cycle (amenorrhea), especially for those with low body fat [24,29]. Previous studies have reported body fat percentages ranging from 20–23% in female fighters. [30,31] The use of oral contraceptives helps regulate the menstrual cycle and stabilize hormonal fluctuations [32]. The effects of the menstrual cycle on athletic performance are mixed [33]. In runners, no effects on performance were reported despite changes in fat mass and body mass [32]. However, decreases in athletic performance were reported in female athletes experiencing amenorrhea [26,28]. It is not entirely clear if these performance changes are directly related to the disruptions in menstrual cycle or factors contributing to amenorrhea (low energy availability, high levels of stress). Changes in body composition, fluid retention, and gastric emptying are reported in relation to hormonal changes [27,32]. Although these changes appear to have minimal impact on athletic performance, they could impact RWL in female UFC athletes.

Despite these clear differences, strategies used during weight cuts are often the same for male and female fighters [8,34–36]. A better understanding of these practices and recovery protocols is imperative to the health and safety of all females participating in MMA. Thus, the primary aim of this study is to provide descriptive information on professional UFC fighters competing in all female weight divisions (strawweight (115 lbs/52.2 kg): flyweight (125 lbs/56.7 kg); bantamweight (135 lbs/61.2 kg); featherweight (145 lbs/65.8 kg)). The researchers hypothesize changes in weight at all time points (72 hrs. pre-weigh-in, 48 hrs. pre-weigh-in, 24 hrs. pre-weigh-in, official weigh-in, and 24 hrs. post-weigh-in) will be similar among all weight divisions.

## 2. Methods

Participants were professional female MMA athletes competing for the UFC organization. Participants competed at strawweight (115 lbs/52.2 kg), flyweight (125 lbs/56.7 kg), bantamweight (135 lbs/61.2 kg), and featherweight (145 lbs/65.8 kg) weight divisions. Data from UFC events occurring between 2020 and 2022 was gathered by the UFC Performance Institute (UFC-PI). Self-reported weights were obtained at five separate time points: 72 hrs. pre-weigh-in, 48 hrs. pre- weigh-in, 24 hrs. pre-competition, official weigh-in, and 24 hrs. post-weigh-in. Recorded weights were supervised by each athlete’s team and reported immediately after weigh-in to UFC-PI staff. All pre- and post-fight weights were obtained using the same make/model scale which were calibrated based on the “official” scale. Official weigh-ins were obtained using a commission-managed beam or digital scale and publicly announced. Athletes that did not compete were excluded from the study. Athletes competing multiple times between 2020 and 2022 were treated as individual data points. Athletes who missed weight or did not officially weigh-in were excluded from the data set. This study of de-identified data was approved by the University Institutional Review Board (IRB). All athletes provided consent to weigh-in and were deemed physically healthy for competition.

### 2.1. Statistical analysis

Descriptive statistics (means and standard deviations) were calculated for age, height, and fight weight, for each weight class. Female weight divisions included the following from lightest to heaviest fighters: strawweight, flyweight, bantamweight, featherweight. Body weight and percent weight reduction change were calculated for each female weight class at 72 hours, 48 hours, 24 hours prior to competition and at weigh-in. Then weight regain, considered a rehydration protocol, was assessed relative to the fighter’s weight the day of the fight, and labeled as post-weigh-in weight or percent weight change. Mixed effects models were used to analyze the influence of the fixed (weight class) and random (Days prior to and day after (fight day) weigh-in) effects on the dependent variables. Mixed effects model analysis was necessary due to the interdependence of the observations within the groups [9,37–39]. Post hoc analyses were complete with Bonferroni test for the fixed effects and pairwise comparisons for the random effects. All statistics were analyzed using IBM SPSS 28.0 (IBM Inc., Armonk, NY). The criterion for statistical significance was set a priori at *p* ≤0.05.

## 3. Results

### 3.1. Physical characteristics

**Figure 1.**
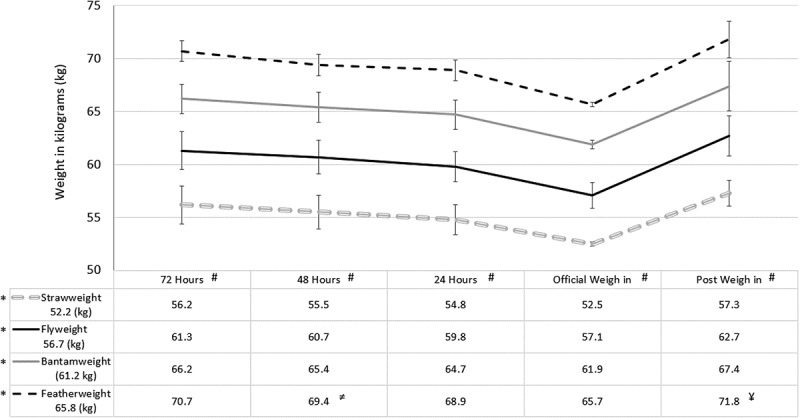
Weight class by weigh-in day fluctuations in body weight in kilograms.

Physical characteristics including age and height were calculated for all female fighters by weight class (Table 1). Absolute weight changes for each group are shown for each day (72 hrs. pre, 48 hrs. pre, 24 hrs. pre, weigh-in, and post weigh-in) in Figure 1.

Data are means in kilograms.

*Indicates significantly different than all other weight classes throughout, *p* < .001.

^#^Indicates significantly different than each weigh-in day throughout, *p* < .001 for all.

^≠^Indicates no difference from 24 hours before for featherweight group only, *p* ≥ .447.

^¥^Indicates no difference from 72 hours before for featherweight group only, *p* ≥ .721.

**Table 1.** Age and height for all fighters by weight class.

Weight Class	Upper Weight Limit	Age (years)	Height (cm)
Strawweight (*n* = 48)	115 lbs. (52.2 kg)	29.5 ± 4.0	161.6 ± 4.5
Flyweight (*n* = 52)	125 lbs. (56.7 kg)	30.7 ± 4.4	165.0 ± 4.4
Bantamweight (*n* = 28)	135 lbs. (61.2 kg)	32.1 ± 5.3	169.0 ± 4.5
Featherweight (*n* = 5)	145 lbs. (65.8 kg)	31.2 ± 1.3	172.2 ± 6.1

Data are presented as means ± standard deviation.

### 3.2. Weight changes by weight class (fixed effects) for each weigh-in day (random effects)

There was no significant weight class by weigh-in day interaction (*F* = 1.444, *p* = .142) for weight change. The fixed effects analyses revealed there was a significant main effect of weight class on weight changes throughout the five-day weigh-in process (*F* = 456.3, *p* < .001, Figure 1). The random effects analyses revealed there was a significant main effect of weigh-in day on weight changes (*F* = 280.7, *p* < .001, Figure 1). Weight differed between weight divisions and changed in a similar fashion throughout the 5-day weigh-in period leading up to a fight.

### 3.3. Percent weight changes by weight class (fixed effects)

The fixed effects analyses revealed there was no significant main effect of weight class on percent weight changes (*F* = .479, *p* = .697, Table 2) throughout the 5-day weigh-in period.

**Table 2.** Percent weight changes by weight class (fixed effects) in female fighters.

Average percent (%) weight change from 72 hours pre to post weigh-in rehydration weight	
Strawweight	−1.7 ± 0.2
Flyweight	−1.9 ± 0.1
Bantamweight	−1.6 ± 0.2
Featherweight	−1.7 ± 0.5

Data are presented as means ± standard deviation.

No difference between weight class, *p* > .697 for all.

### 3.4. Percent weight changes each weigh-in day (random effects)

The random effects analyses revealed there was a significant main effect of weigh-in day on percent weight changes (*F* = 488.9, *p* < .001). Post hoc analysis revealed there were significant differences between every weigh-in day (*p* < .001 for all, Table 3).

**Table 3.** Percent weight changes each weigh-in day (random effects).

Percent (%) Weight Change	
72 Hours Before	−6.6 ± 2.1^a,b,d^
48 Hours Before	−5.4 ± 1.9^a,c,d^
24 Hours Before	−4.3 ± 1.8^b,c,d^
Post Weigh-in	9.5 ± 3.2^a,b,c^

Data are presented as means ± standard deviation.

^a^Significantly different from 24 hours pre weigh-in, *p* < .001.

^b^Significantly different from 48 hours pre weigh-in, *p* < .001.

^c^Significantly different from 72 hours pre weigh-in, *p* < .001.

^d^Significantly different from post weigh-in, *p* < .001.

### 3.5. Weight class (fixed effects) by weigh-in day (random effects) for percent weight change

There was no significant weight class by weigh-in day interaction (*F* = 0.382, *p* = 0.943, Table 4) for percent weight change. Regardless of weight class, female fighters experience similar weight changes across each day prior to a fight.

**Table 4.** Percent weight changes by weight class (fixed effects) for each weigh-in day (random effects) for female fighters.

Weight Class	72 hours before	48 hours before	24 hours before	Post Weigh-in
Strawweight^*,#,%,x^	−6.5 ± 1.9	−5.4 ± 1.8	−4.2 ± 1.7	9.2 ± 3.2
Flyweight^*,#,%,x^	−6.8 ± 2.5	−6.0 ± 2.0	−4.6 ± 2.1	9.8 ± 3.7
Bantamweight^*,#,%,x^	−6.4 ± 1.7	−5.2 ± 1.6	−5.1 ± 1.8	9.4 ± 2.8
Featherweight ^*,x^	−6.6 ± 2.3	−5.0 ± 2.1	−4.2 ± 2.0	8.9 ± 1.9

Data are presented as means ± standard deviation.

There were no significant differences between weight classes at 72, 48, 24 hours before and post weigh-in, *p* ≥ .943 for all.

*Significantly different from 72 hours pre weigh-in, *p* < .001.

^#^Significantly different from 48 hours pre weigh-in, *p* ≤ .006.

^%^Significantly different from 24 hours pre weigh-in, *p* ≤ .003.

^x^Significantly different from post weigh-in weight, *p* ≤ .006.

## 4. Discussion

Knowledge about rates of weight loss in female fight sport athletes utilizing RWL techniques is essential to providing evidence-based recommendations that enhance performance. Despite the exponential growth of female athletes competing in the UFC, few studies have elucidated the effects of weight loss in this population. To the authors' knowledge, this is the first study to provide descriptive statistics on the changes of weight loss in female athletes competing in the UFC. Changes existed in body mass across 5 time points (72 hrs. pre-weigh-in, 48 hrs. pre-weigh-in, 24 hrs. pre-weigh-in, official weigh-in, and 24 hrs. post-weigh-in) in all female weight divisions (i.e. strawweight (115 lbs/52.2 kg): flyweight (125 lbs/56.7 kg); bantamweight (135 lbs/61.2 kg); featherweight (145 lbs/65.8 kg)).

The greatest change in weight was reported between official weigh-in and competition (Table 3). This is most likely the result of rehydration practices and glycogen repletion. Proper rehydration and weight regain practices are associated with better performance outcomes [10,40]. The average weight regained among participants was 9.5 ± 3.2%. These findings are similar to those reported by Peacock et al. [9] but greater than the weight regain reported by previous studies [10,40–42]. Differences in discipline, time between official weigh-in and competition and/or RWL strategies could account for the variability.

All participants lost a significant amount of body mass between each time point. This study observed decreases in body mass ranging from 4.5–6.6% of body mass. Similar findings have been reported in studies focusing on MMA athletes which report weight losses ranging from 3–10% of body mass [5,10,12,19,20]. Peacock et al. [9] reported similar percentages loss in body mass at all time points specifically in UFC fighters. Other disciplines of fight sports tend to report smaller changes in body mass (2–6%) [19,43], however our findings are still like the upper end of the reported range. It is suggested that MMA athletes experiences the greatest changes in body mass [19]. In female wrestlers, Viveiros et al. [44] reported similar changes in body mass of 6.3%. Unlike the previously mentioned studies, Drid et al. [35], reported a greater yet non-significant change in body mass pre and post competition in male sambo athletes compared to female sambo athletes. The findings of this study are similar to changes in female sambo fighters. Kazemi et al. [45] reported gender differences in bantamweight and light middleweight divisions but not heavyweight divisions in adolescent Taekwondo athletes. Although our findings are consistent with those in the literature, the absence of male participants prevents a side-by-side comparison of changes in body mass.

No statistical differences were reported in rates of weight loss between weight divisions. Our findings differ from those a previous study highlighting weight loss prior to competition in UFC . [9] Peacock et al. reported significant differences in weight loss among weight classes compared to the heavyweight division. Fighters in the featherweight division experienced the greatest change in weight while the light heavyweight division exhibited the least change in weight. [9] Some weight class divisions, such as heavyweight, do not need to cut much weight compared the other divisions.

Participants in all weight classes significantly decreased their weight at each time point suggesting the use of various strategies to induce RWL. These findings are consistent with other studies highlighting the prevalence of RWL practices in fight sports [8,11,17,34–36]. It is estimated that as many as 95% of fight sports athletes engage in RWL practices days prior to competition [5,8,11,19]. It is worth noting that none of these studies reported gender differences in RWL techniques used by fight sport athletes. Unlike the aforementioned studies, this study did not report any of the RWL practices utilized by subjects.

## 5. Conclusion

RWL is a routine practice in female fighters competing in the UFC. Our findings suggest that females engaged in these practices lost weight in a similar fashion irrespective of weight class. Based on the available literature, the body mass lost (4.3–6.6%) and regained (8.9–9.8%) during this process are comparable to other fight sport athletes.
